# Ophthalmic branch radiofrequency thermocoagulation for atypical trigeminal neuralgia:a case report

**DOI:** 10.1186/s40064-015-1624-0

**Published:** 2015-12-24

**Authors:** Shibin Du, Xiaoliang Ma, Xiaoqin Li, Hongjie yuan

**Affiliations:** Department of Anesthesiology and Pain Management, Xijing Hospital, Fourth Military Medical University, 710032 Xian, China

**Keywords:** Trigeminal neuralgia, Upper eyelid, Ophthalmic branch, Radiofrequency thermocoagulation

## Abstract

**Background:**

Trigeminal neuralgia is an intense neuralgia involving facial areas supplied by trigeminal nerve. The pain is characterized by sudden onset, short persistence, sharp or lancinating. Trigeminal neuralgia commonly affects frontal areas, infraorbital or paranasal areas, mandibular areas and teeth. While Trigeminal neuralgia affecting merely the upper eyelid is rare. Here we report a case of atypical Trigeminal neuralgia confined to the upper eyelid. The patient was pain free during the follow-up period of 6 months after unusual ophthalmic branch radiofrequency thermocoagulation.

**Case presentation:**

A 55-year-old female patient was diagnosed as primary trigeminal neuralgia involving the right upper eyelid. As the pain could not be controlled by drug therapy, peripheral nerve branch radiofrequency thermocoagulation was recommended. A combination of infratrochlear, supratrochlear and lacrimal radiofrequency thermocoagulation was implemented in this case. The point where the bridge of the nose abuts the supraorbital ridge and the point slightly above the lateral canthus along outer border of the orbit were selected respectively as the puncture sites. After positive diagnostic test, radiofrequency thermocoagulation of the above-mentioned nerve branches was performed respectively. The patient was pain free immediately after the treatment and during the follow-up period of 6 months.

**Discussion:**

Trigeminal neuralgia is a common severe and chronic facial neuralgia which requires accurate diagnosis and effective therapy. With typical clinical symptoms, normal neurological signs, normal CT and MRI findings, the patient was diagnosed as classic trigeminal neuralgia. As the patient was drug resistant, some invasive treatments were considered. Peripheral branch neurolysis was chosen for its minimal invasiveness, convenience, low risk and not affecting further invasive treatments. According to the anatomic data and the diagnostic test results, infratrochlear, supratrochlear and lacrimal nerve were responsible, therefore, an unusual combination of infratrochlear, supratrochlear, and lacrimal radiofrequency thermocoagulation was implemented for this patient.

**Conclusions:**

Radiofrequency thermocoagulation is an effective treatment option for trigeminal neuralgia. Peripheral branch radiofrequency thermocoagulation for trigeminal neuralgia should be considered preferentially due to its minimal invasiveness and convenience. Furthermore, as the sensory innervation of the upper eyelid is complex, the knowledge of peripheral distribution of trigeminal nerve is essential.

## Background

Trigeminal neuralgia (TN) is a common disease mainly occurs in the elderly. It is a severe facial pain typically featured by paroxysmal onset, short persistence, sharp, stabbing or electric shock like in the regions innervated by certain branches of the trigeminal nerve (Headache Classification Committee of the International Headache S 2013). Attacks can be triggered by washing face, speaking, brushing, eating and any contact with certain part of face. Clinically, frontal areas, infraorbital or paranasal areas, mandibular areas and teeth are the mostly affected areas in TN. Here we report a rare case of drug-resistant TN affecting only the upper eyelid. Percutaneous radiofrequency trigeminal neurolysis is an effective, safe, and minimal-invasive treatment for those refractory to drug therapy. However, the sensory innervation of the upper eyelid is complex. After review of the anatomic data and the diagnostic nerve block, we applied neurolysis to infratrochlear, supratrochlear and lacrimal nerves respectively in this case.

## Case presentation

Here was a 55-year-old female patient with episodic stabbing pain on the right superior eyelid for 2 years. The VAS score was eight. The onset time was about 1 minute and the remission period was completely pain free. The pain could be triggered by daily activities like smiling, washing face, speaking and eating. Any contact with the upper eyelid could lead to pain outbreak. Apart from the allodynia on the skin of upper eyelid, no abnormality was found in neurological examinations. Also, the ophthalmologic exams including funduscopy, intraocular pressure, visual field testing was normal. The head CT and MRI scan were also normal. The patient had no history of surgical operation and trauma, no history of diabetes mellitus, high blood pressure, chronic heart diseases, hepatitis, and some other relevant chronic diseases. The patient was diagnosed as primary TN.

The treatment was initiated by oral carbamazepine. The pain could be relieved by carbamazepine 200 mg daily at first. Gradually increasing dose of carbamazepine was reported during the medication period. About 2 weeks prior to the treatment, the pain could not be controlled by carbamazepine 800 mg daily, and dizziness and vertigo developed simultaneously.

**Fig. 1:**
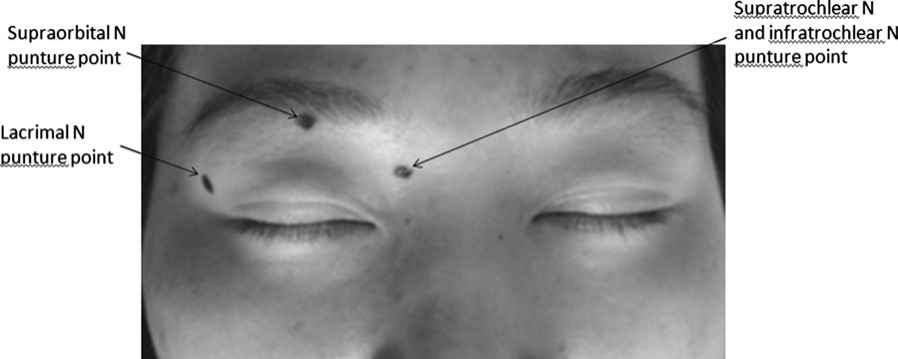
Respective puncture *points* of the nerves innervating the *upper* eyelid

Peripheral branch neurolysis was planned. Firstly, right supraorbital nerve block was implemented as a diagnostic test. After identifying the supraorbital notch through palpation, a 25-gauge needle was inserted at the level of supraorbital notch. 0.5 ml 1 % lidocaine was injected slowly. Ten minutes later, hypoaesthesia in the skin of the forehead instead of the upper eyelid was observed, and the pain continues. This result indicated that supraorbital nerve was not involved. Then, diagnostic blocks to lacrimal, infratrochlear and supratrochlear nerves were successively performed. The puncture point slightly above the lateral canthus along outer border of the orbit was selected for the lacrimal nerve block. As infratrochlear and supratrochlear exiting points were located closely together, we chose the point where the bridge of the nose abuts the supraorbital ridge as the single puncture point (Fig. 1). The patient was put in supine position while eyes firmly closed. After disinfection, a 25-gauge needle was punctured at the lacrimal nerve point described above. The needle was slid over the edge of the orbit, and advanced about 4 mm along the lateral wall of the orbit. With no aspiration of blood, 0.3 ml 1 % lidocaine was injected slowly. Five minutes later, hypoesthesia was found on the lateral part of the upper eyelid, while the medial part remained allodynic. Then the infratrochlear and supratrochlear nerve blocks were performed. The needle was inserted in the previously mentioned point, just slid over the edge, with the same depth along the medial wall. The same amount of lidocaine was injected slowly. The whole upper eyelid and adjacent areas were found hypoesthetic. The patient was completely pain free for the whole day. Then lacrimal, infratrochlear and supratrochlear nerves radiofrequency thermocoagulation (RT) was planned the next day. With supine position, the skin of upper eyelid and adjacent areas was disinfected. From the two puncture points above-mentioned, a 22 G radiofrequency needle with a working tip of 4 mm was slowly inserted successively. Slide over the edge of the orbit and advance about 4 mm. Repositioning the needle slightly to the point where sensory test stimulation at 0.3 mA and 50 Hz could evoke obvious paresthesia in the periocular region. As for infratrochlear and supratrochlear nerves RT, with the aim to ablate both the two nerves, redirect the needle tip superiorly and inferiorly along the medial wall of the orbit to make additional ablation. After local anaesthesia, RT with tip temperature of 80 °C and duration of 80 s was performed.

The patient was discharged after 2 h observation. There were no complications, except for hypoesthesia in the upper eyelid and eyelid swelling for 2 days. The pain was completely relieved within the follow-up period of 6 months without any medication.

## Discussion

Trigeminal neuralgia is a common disease causing facial pain. The reported prevalence of TN is 4.3 per 100,000 (Obermann and Katsarava 2009). And also due to the severity and choronicity of the pain, patients’ daily lives are severely affected. It is reported that TN patients often have a certain extent depression, with high levels of anxiety during acute attacks. Therefore accurate diagnosis and effective therapy of the disease are essential.

As the International Classification of Headache Disorders reports, classic TN is usually resulted from microvascular compression of trigeminal nerve root. While symptomatic TN can be caused by other factors, such as vascular disorders, tumors, demyelination resulted from multiple sclerosis (Headache Classification Committee of the International Headache S 2013). With typical clinical symptoms, together with normal neurological signs, normal CT and MRI findings, diagnosis of symptomatic TN was ruled out, the patient was diagnosed as classic TN. However, TN cases affecting the first branch only account for 4 % (Vorenkamp 2013) and TN cases affecting merely the upper eyelid are never reported. Therefore it was a rare case of classic TN affecting only the upper eyelid.

Although some kind of eye diseases may have pain symptoms, it could be differentiated by the characteristics of pain. The eye diseases induced pain is dull and persistent, while TN is sharp and transient attacking. Cluster headache is another severe facial pain which should be distinguished. The pain usually locates in retro-orbital and temporal areas with paroxysmal occurrence of 15–180 minutes, and accompanied by parasympathetic symptoms like ptosis, pupil constriction, facial flushing, conjunctival conjestion and even vomiting (Voiticovschi-Iosob et al. 2014).

The treatment of TN should be initiated by oral medication, due to its none-invasiveness, low cost and high effective rate. Some invasive treatments are only suitable for those refractory to oral medication. Those include peripheral nerve branch neurolysis, trigeminal ganglion RT, trigeminal nerve balloon compression, sterostatic radiosurgery Gamma knife and microvascular decompression. In this case, as oral medication was ineffective, further invasive treatment is considered. Trigeminal ganglion RT was not considered, as severe complications may happen in treating TN of branch V1, such as corneal hypoesthesia and neuroparalytic keratitis (van Loveren et al. 1982; Huibin et al. 2009). Balloon compression may have a risk of masticatory muscle dysfunction temporarily (Lopez et al. 2004). Stereotactic radiosurgery is relatively costly and has slow onset of action and usually takes about 1 month before the onset of pain alleviation (Zakrzewska and Linskey 2014). Heinskou etc. reported an accelerated cross-speciality management program which proved to be feasible and of high quality. They argued that for the classical TN patients refractory to oral medication, microvascular decompression should be preferentially applied if microvascular compression was indicated in MRI, the second choice should be balloon compression (Heinskou et al. 2015). As for our patient, no neurovascular contact was found in the MRI. Also considering peripheral nerve RT is safe, minimal invasive, convenient and not affecting possible further surgical treatments, the peripheral nerve RT was cautiously applied in this case.

Supraobital, infraorbital, and mental nerve blocks are the three commonly adopted peripheral nerve branch block in treatment of TN. These conventional treatments were ineffective in this case as the diagnostic test verified. According to the anatomic data (Standring 2008), the supraorbital and supratrochlear nerve, both originated from the frontal nerve, innervate part of the upper eyelid skin. So does the infratrochlear nerve which derived from nasociliary nerve, a branch of ophthalmic nerve. Lacrimal nerve, originated directly from the ophthalmic nerve, after travelling through the lacrimal gland, innervates the lateral part of the superior eyelid skin. These four nerves leave the orbit through respective exiting points supplying the upper eyelid skin. Due to the negative result of diagnostic supraorbital nerve block, we speculated that the supraorbital nerve does not give sensory innervations to the upper eyelid in this case. This also indicates the necessity of diagnostic nerve block before treatment. In this patient, the anatomic data together with the diagnostic test confirmed that infratrochlear, supratrochlear and lacrimal nerve were responsible. Therefore, a combination of infratrochlear, supratrochlear, and lacrimal neurolysis was used for this patient.

Furthermore, there are some somatic and autonomic motor nerves in the orbit, such as the abducens nerve innervating the lateral rectus, the trochlear nerve innervating superior oblique, the oculomotor nerve to the remaining extraocular muscles, and also the lacrimal nerve, which carries postganglionic parasympathetic nerve fibres from the pterygopalatine ganglion supplying the lacrimal gland (Standring 2008). With the nerve stimulation function and controlled tip temperature, RT can generate accurate and definite volume of ablation around the working end. This ensures avoiding injury to these important nerves.

## Conclusions

Our literature search identified only one article involving lacrimal nerve block (Pareja and Cuadrado 2013). It was used as diagnostic test for what is called lacrimal neuralgia. This case report demonstrated the importance of knowledge of trigeminal nerve anatomy and priority of peripheral branch neurolysis due to its effectiveness, minimal invasiveness and noninterference with possible further treatments.

## Consent

Written informed consent was obtained from the patient for publication of this case report.
